# Arabidopsis nonhost resistance gene *PSS1* confers immunity against an oomycete and a fungal pathogen but not a bacterial pathogen that cause diseases in soybean

**DOI:** 10.1186/1471-2229-12-87

**Published:** 2012-06-13

**Authors:** Rishi Sumit, Binod B Sahu, Min Xu, Devinder Sandhu, Madan K Bhattacharyya

**Affiliations:** 1 Department of Agronomy, Iowa State University, Ames, IA, 50011, USA; 2 Molecular Cellular and Developmental Biology Interdepartmental Graduate program, Iowa State University, Ames, IA, 50011, USA; 3 Department of Plant and Microbial Biology, UC Berkeley, Berkeley, CA, 94720, USA; 4 Biology Department, University of Wisconsin, Stevens Point, Wisconsin, 54481, USA

**Keywords:** *Phytophthorasojaes*usceptible (*pss1*), Sequence-based polymorphic (SBP) marker, *Fusarium virguliforme*, *Phytophthora sojae*, *Pseudomonas syringae* pv. *glycinea*

## Abstract

**Background:**

Nonhost resistance (NHR) provides immunity to all members of a plant species against all isolates of a microorganism that is pathogenic to other plant species. Three *Arabidopsis thaliana PEN* (penetration deficient) genes, *PEN1, 2* and *3* have been shown to provide NHR against the barley pathogen *Blumeria graminis* f. sp*. hordei* at the prehaustorial level. Arabidopsis *pen1-1* mutant lacking the *PEN1* gene is penetrated by the hemibiotrophic oomycete pathogen *Phytophthora sojae,* the causal organism of the root and stem rot disease in soybean. We investigated if there is any novel nonhost resistance mechanism in Arabidopsis against the soybean pathogen, *P. sojae.*

**Results:**

The *P.sojae*susceptible (*pss*) *1* mutant was identified by screening a mutant population created in the Arabidopsis *pen1-1* mutant that lacks penetration resistance against the non adapted barley biotrophic fungal pathogen, *Blumeria graminis* f. sp. *hordei.* Segregation data suggested that *PEN1* is not epistatic to *PSS1*. Responses of *pss1* and *pen1-1* to *P. sojae* invasion were distinct and suggest that PSS1 may act at both pre- and post-haustorial levels, while PEN1 acts at the pre-haustorial level against this soybean pathogen. Therefore, *PSS1* encodes a new form of nonhost resistance. The *pss1* mutant is also infected by the necrotrophic fungal pathogen, *Fusarium virguliforme,* which causes sudden death syndrome in soybean. Thus, a common NHR mechanism is operative in Arabidopsis against both hemibiotrophic oomycetes and necrotrophic fungal pathogens that are pathogenic to soybean. However, *PSS1* does not play any role in immunity against the bacterial pathogen, *Pseudomonas syringae* pv*. glycinea,* that causes bacterial blight in soybean. We mapped *PSS1* to a region very close to the southern telomere of chromosome 3 that carries no known disease resistance genes.

**Conclusions:**

The study revealed that Arabidopsis *PSS1* is a novel nonhost resistance gene that confers a new form of nonhost resistance against both a hemibiotrophic oomycete pathogen, *P. sojae* and a necrotrophic fungal pathogen, *F. virguliforme* that cause diseases in soybean. However, this gene does not play any role in the immunity of Arabidopsis to the bacterial pathogen, *P. syringae* pv*. glycinea*, which causes bacterial blight in soybean. Identification and further characterization of the *PSS1* gene would provide further insights into a new form of nonhost resistance in Arabidopsis, which could be utilized in improving resistance of soybean to two serious pathogens.

## Background

Plants are exposed to an innumerable number of pathogenic organisms on a daily basis. However, because of immunity mechanisms only a few pathogens can infect and cause diseases in a particular crop species. One of the less understood immunity mechanisms is nonhost resistance (NHR), exhibited by all members of a plant species against non adapted pathogens [1,2]. The main NHR mechanisms were thought to be 1) incompatibility of non adapted pathogen with the physiology of nonhost plants and 2) inability of non adapted pathogens to overcome the plant defenses [3]. The first gene known to confer *Arabidopsis* NHR against a non adapted bacterial pathogen, *Pseudomonas syringae* pv. *phaseolicola,* is *NONHOST1* (*NHO1*) which encodes a glycerol kinase [4,5]. *NHO1* has also been shown to play an important role in the expression of gene-specific resistance against a bacterial pathogen [4].

NHR acts in two layers against the biotrophic fungal pathogens [6,7]. The first layer of NHR suppresses the invasion by non adapted pathogens at the pre-haustorial level. Three NHR genes, *PEN1, PEN2* and *PEN3*, required for penetration resistance of Arabidopsis against the non adapted barley biotrophic fungal pathogen, *Blumeria graminis* f. sp. *hordei* have been isolated [6-8]. These genes act at the prehaustorial stage of the pathogen invasion [9]. *PEN1* encodes a soluble N-ethylmalemide sensitive attached receptor (SNARE) protein, which is involved in vesicle fusion and exocytosis of toxic compounds to the pathogen infection sites [8]. *PEN2* encodes a glycosyl hydrolase, which has been localized to the peroxisomes [6]. *PEN3* encodes an ATP-binding cassette (ABC) protein of the plasma membrane [7]. Cytological studies have demonstrated that *PEN2* and *PEN3* work together to generate and transport toxic chemicals into the infection sites [10]. The first layer of NHR prevents the biotrophic fungal pathogens from penetration and development of feeding structures, haustoria. Fungal pathogens that overcome the first layer of NHR encounter a post-haustorial defense mechanism. Some of the genes involved in the second layer of NHR in Arabidopsis are *EDS1, PAD4* and *SAG101* that are involved in plant defenses [6]. Downstream antagonistic defense pathways regulated by salicylic acid (SA) and the jasmonic acid (JA) are activated upon infection with biotrophic and necrotrophic pathogens, respectively [11]. SA and JA pathways are shown to be involved in the expression of nonhost resistance against the cowpea rust, *Uromyces vignae*, in Arabidopsis [12]. Similarly, studies of mutants lacking *PEN1, 2,* and *3* established that SA and JA pathways are also involved in the expression of nonhost resistance in Arabidopsis against the soybean pathogen *Phakopsora pachyrhizi* that causes the Asian soybean rust [13].

Recognition of pathogen associated molecular patterns (PAMPs) of non adapted pathogens by PAMP recognition receptors (PRRs) triggers the PAMP-triggered immunity (PTI) in nonhost species [14]. Recent studies have shown that PTI plays a major role in NHR [15]. Both chemical and physical barriers induced by PTI restrict non-adapted pathogens from invading nonhost species. Physical barriers include callose deposition at the infection sites and preformed barriers such as waxy coating on leaves. Chemical barriers include deposition of various reactive oxygen species (ROS) such as hydrogen peroxide and phenolic compounds at the infection site [16,17].

The plant responses to pathogenic invasions can be classified into two broad groups, PTI and the effector-triggered immunity (ETI) activated by strain-specific effectors. Both PTI and ETI play roles in providing nonhost resistance of plant species against non-adaptive or nonhost pathogens. It is speculated that PTI and ETI play an increasingly major and a minor role, respectively, in conferring nonhost resistance as the evolutionary distance between the nonhost and the nonhost pathogen species widens [18]. Conversely, ETI and PTI play an increasingly major and a minor role, respectively, in expression of nonhost resistance as the evolutionary distance between the nonhost and nonhost pathogens reduces.

Soybean (*Glycine max* L. *Merr*.) is one of the most important oil seed crops, a major source of livestock feed and an important biodiesel crop. Unfortunately, soybean is also a host of many pathogens that cause several serious diseases resulting in an estimated annual yield loss of $2.26 billion dollars [19]. In the United States, the estimated annual soybean yield losses just from the oomycete pathogen, *P. sojae*, have been valued to be over 300 million dollars [19]. Although various *Rps* (resistance to *P. sojae*) genes are utilized in generating *Phytophthora* resistant soybean cultivars [20,21], resistance conferred by these genes is effective only against a set of *P. sojae* races and is not durable. Partial resistance governed by quantitative trait loci (QTL) confers broad-spectrum resistance against *P. sojae* races in soybean. However, the level of partial resistance is not adequate enough to prevent significant crop losses [22]. Thus, it is essential to identify and use NHR mechanisms to provide soybean with broad-spectrum and durable resistance against this pathogen. As a first step towards achieving this goal, we have applied a forward genetic approach to identify and map the *Arabidopsis thaliana* NHR gene, *PSS1,* which provides resistance against the oomycete pathogen *P. sojae*. *PSS1* is also required for immunity of Arabidopsis against the fungal pathogen, *Fusarium virguliforme* that causes the sudden death syndrome (SDS) in soybean.

## Results

### Arabidopsis *pen1-1* mutant, but not *nho1* mutant, is penetrated to single cells by the soybean pathogen *P. sojae*

**Figure 1:**
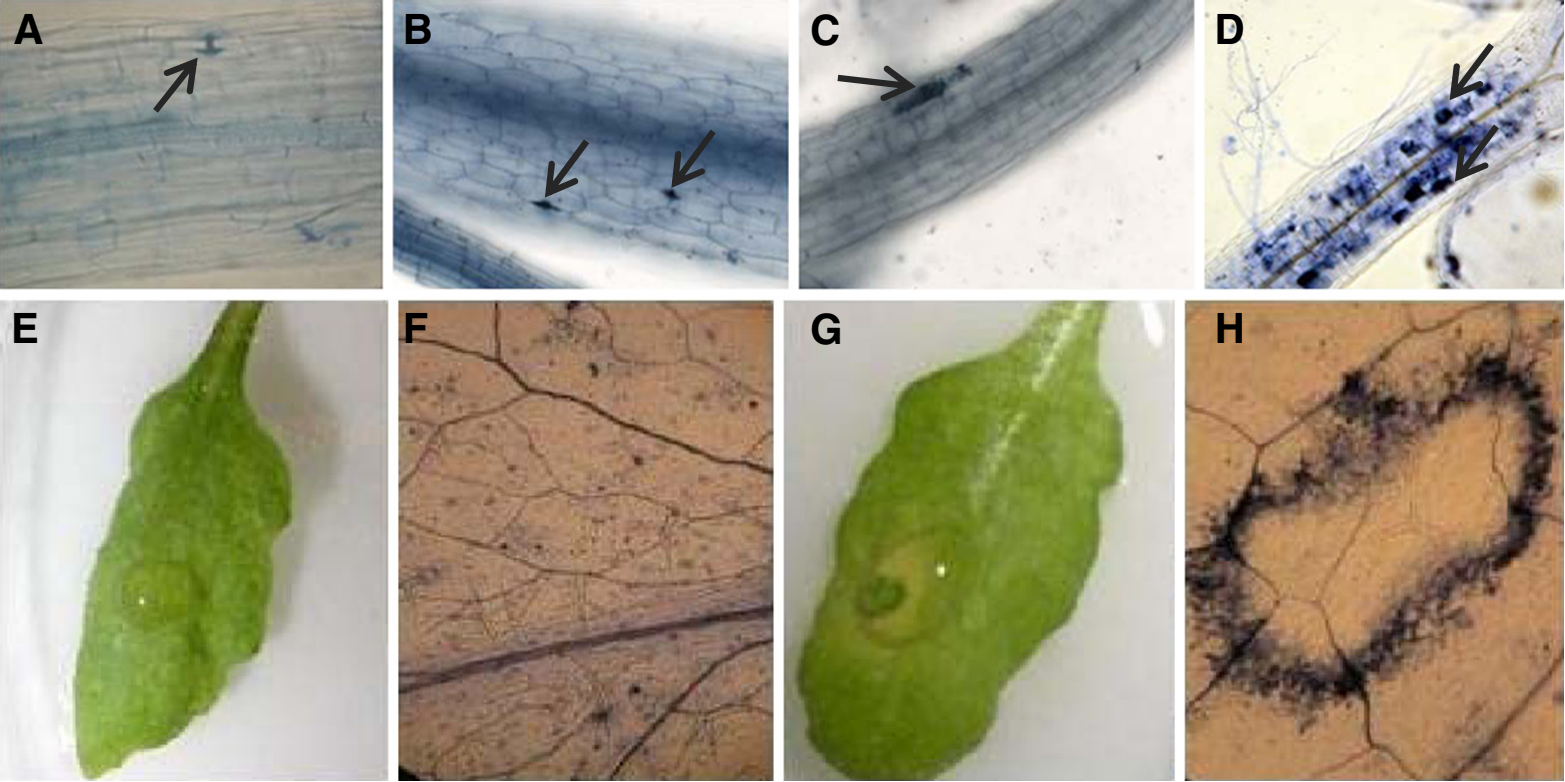
**Identification of the *pss1 *mutant A,** Columbia-0 and **B,***nho1* seedlings were not penetrated by *P. sojae*. **C,** single cells of *pen1-1* were penetrated by *P. sojae* and cell death occurred following penetration. **D,** The *pss1* mutant showed penetration and colonization by *P. sojae*. Images shown in A, B, C and D were taken at 100X magnification. Arrows in A and B show failed attempts of penetration by germinating zoospores. Arrows in C and D show the cell death caused by penetrating hyphae. **E** and **F,** macroscopic and microscopic responses of *pen1-1* following *P. sojae* infection; **G** and **H,** macroscopic and microscopic responses of *pss1* leaf following *P. sojae* infection. Images of F and H were taken at 50X magnification. The photographs show representative results obtained from three independent experiments. Microscopic images of A, B, C, D, F and H were taken following staining of infected tissue samples with trypan blue.

Arabidopsis *nho1* and *pen1-1* mutants are defective in NHR mechanisms against the bacterial pathogen, *Pseudomonas syringae* pv. *phaseolicola*[5] and the powdery mildew fungus, *Blumeria graminis* f. sp. *hordei*[8], respectively. We investigated if the soybean pathogen *P. sojae* infects either of the two mutants. Ten-day-old seedlings grown in autoclaved double distilled water were inoculated with *P. sojae* zoospore suspensions and incubated for two days in the dark at 22°C. The inoculated seedlings were then stained with trypan blue dye and observed under a light microscope [23]. The pathogen did not penetrate either the wild-type ecotype Columbia-0 (Col-0) or the *nho1* mutant (Figures 1A and B). *P. sojae* however penetrated single cells in *pen1-1* (Figure 1C). These results indicated that in the *pen1-1* mutant, the pre-haustorial NHR against *P. sojae* is compromised*.*

### Identification of *Phytophthorasojaesusceptible* (*pss*) putative mutants

We mutagenized *pen1-1,* compromised in pre-invasive immunity against *P. sojae*, with ethyl methane sulfonate (EMS) to identify mutants that are compromised in post-invasive immunity mechanisms. Over 3,500 M_1_ plants were planted and M_2_ seeds of these plants were harvested individually. Three hundred and seventy-nine randomly selected M_2_ families were grown to score for the chlorophyll mutants, a marker for determining the extent of EMS-induced mutation. About 5% of the families segregated for albino plants (Additional file 1), which suggested that the mutant population contained sufficient random point mutations and was suitable for screening. Approximately ≥ 70 seedlings of each M_2_ family were grown aseptically in 24-well microtiter plates in sterile water at 22°C for 10 days before inoculating with *P. sojae* zoospores. Following inoculation, seedlings were incubated for two days at 22°C in the dark, and then seedlings were stained with trypan blue for identifying putative mutants via staining of dead infected cells [23]. From screening 3,500 M_2_ families, we identified 30 putative mutants that were penetrated by *P. sojae* to multiple cells. The putative mutants were named as *Phytophthorasojae*susceptible 1 (*pss1*) through *pss30*. Subsequently, a detached leaf inoculation technique, previously reported for soybean, was applied in screening the putative mutants to identify the homozygous mutant plants [24]. We have applied a mapping approach in classifying these putative mutants. A homozygous mutant M_4_ family (0.2B_17_I_9_-24) of the putative mutant *pss1* showing complete loss of both pre- and post-haustorial NHR against *P. sojae* was selected. In successive generations, the selected *pss1* mutant family was consistently infected by *P. sojae*. This mutant was phenotypically different from the *pen1-1* because death in the mutant seedlings occurs in multiple cells as compared to in single cells in the *pen1-1* mutant (Figure 1D, E, F, G, H). Although the *P. sojae* zoospores germinated and were able to form appresoria at the infection site, its growth was arrested immediately following germination on wild type Col-0 leaves. The *pen11* mutant showed occasional death in single cells following *P. sojae* infection.

**Figure 2:**
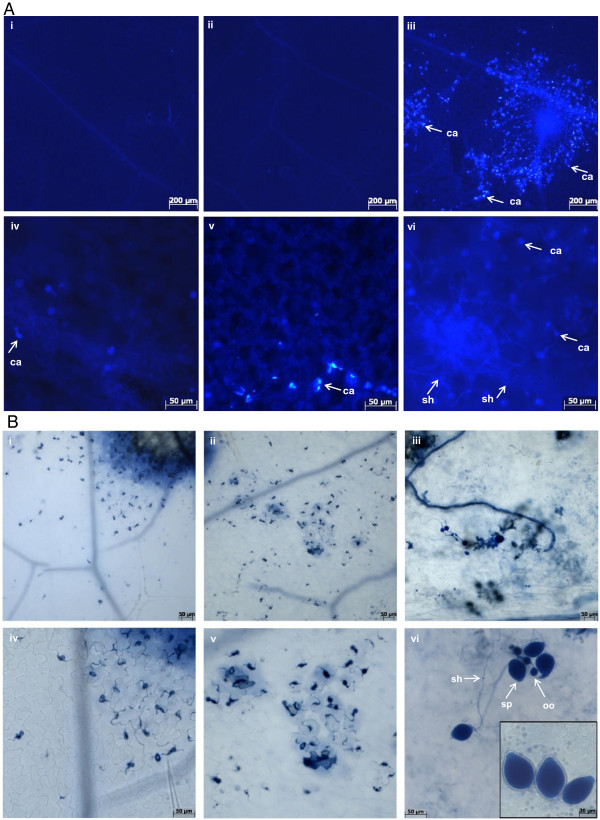
**Responses of the *pss1 *mutant following *P. sojae *infection. A,** Leaves of 21 day old Col-0, *pen1-1* and *pss1* seedlings were inoculated with *P. sojae* zoospores and stained with aniline blue and visualized under a Zeiss Axioplan II compound microscope with ultraviolet epifluorescence [25]. (i) and (iv), Col-0; (ii) and (v), *pen1-1;* and (iii) and (vi), *pss1* leaves that were stained with aniline blue to detect callose deposition 6 hours post inoculation (hpi) with *P. sojae* by epifluorescence of the aniline blue. (i-iii), 50X magnification; and (iv-vi), 200X magnification. Arrows indicate sites of callose deposition (ca) and secondary hyphae (sh). The experiment was repeated twice with similar results. **B**, Leaves of 21 day old Col-0, *pen1-1* and *pss1* seedlings were inoculated with *P. sojae* zoospores and stained with trypan blue and visualized under a Zeiss Axioplan II compound microscope under bright field illumination [23]. (i) and (iv), Col-0; (ii) and (v), *pen1-1;* and (iii) and (vi), *pss1* leaves that were stained with trypan blue to detect cell death and fungal structures 7 days following inoculation with *P. sojae* zoospores. Arrows indicate reproductive structures, oogonia (oo), sporangia (sp) and secondary hyphae (sh), which were visible in infected *pss1* leaves. (i-iii), 100X magnification; and (iv-vi), 200X magnification. The experiment was repeated twice with similar results.

To determine the extent of *P. sojae* growth in infected tissues, detached *pss1* leaves were collected 6 hours post inoculation (hpi) with *P. sojae* zoospore suspensions or treatments with water droplets. Leaves were then stained with aniline blue and the ultraviolet epiflourescence was visualized using a Zeiss Axioplan II compound microscope [25]. Extensive colonization by the pathogen was observed in the *pss1* mutant (Figure 2A). Aniline blue stains the callose deposition and papillae formation and can be used to visualize fungal structures such as runner hyphae [26,27]. Callose deposition and papillae formation have previously been used as markers for attempted penetration by fungal pathogen [7]. Following inoculation with *P. sojae* zoospores, *pss1* leaves showed extensive callose deposition and papillae formation across the infected leaf tissue as compared to *pen1-1* and Col-0 (Figure 2A). Neither callose deposition nor papillae formation was detected in detached leaves that were treated with water droplets (Additional file 2A). At 6 hpi, extensive growth of the secondary hyphae was observed in *P. sojae* infected leaves of *pss1* but not that of Col-0 and *pen1-1* (Figure 2A).

To determine if *P. sojae* became adapted to the Arabidopsis *pss1* mutant, we conducted microscopic study of the diseased lesions of the detached *pss1* leaves 7 days post-inoculation (dpi) with the zoospore suspensions of the oomycete (Figure 2B). We observed enhanced hyphal growth and formation of reproductive structures, sporangia and oogonia on *pss1* leaves (Figure 2B, Additional file 2B). Thus, we conclude that a gene mutated in *pss1* is crucial for pre- and post-invasive nonhost immunity of Arabidopsis against the soybean pathogen, *P. sojae*. We named this gene *PSS1*.

### Arabidopsis ecotypes showed leakiness in their NHR responses to *P. sojae*

Columbia-0 (Col-0) and *Landsberg erecta* (L*er*) are the two most well characterized ecotypes of *Arabidopsis thaliana* for mapping and gene cloning experiments [28,29]. We investigated if the ecotype L*er* was completely immune to *P. sojae* so that it could be crossed to *pss1* for generating mapping populations. However, L*er* showed leakiness in its immune response against *P. sojae* and a significant proportion (12.5%) of the L*er* seedlings were infected by *P. sojae* (Table 1). This result is not very surprising because the Arabidopsis ecotype *L. erecta* has recently been found to show susceptibility to another oomycete pathogen, *Pythium irregulare*[26]. We therefore inoculated 22 *A. thaliana* ecotypes with *P. sojae* zoospores and discovered that ecotypes, Bensheim, Nossen-0 (No-0) and Niederzenz-0 (Nd-0) were completely immune to the pathogen (Table 1). We selected Nd-0 for mapping experiments because it is morphologically similar to Col-0. Furthermore, a few molecular markers polymorphic between Nd-0 and Col-0 were already available [30].

**Table 1:** **Responses of Arabidopsis ecotypes to *P. sojae***

**Seedling Inoculation**				**Leaf Inoculation**		
Ecotypes	^1^Immune	^2^Infected	% Infection	^1^Immune	^2^Infected	% Infection
AUA/Rhon	42	0	0.00	-	-	-
Bensheim	45	0	0.00	-	-	-
Cape Verde-0	24	1	4.00	19	5	20.83
Catania	-	-	-	21	3	12.50
Columbia-0	250	5	1.96	20	1	4.76
Da(1)	-	-	-	17	7	29.17
Ellershausen-0	-	-	-	19	5	20.83
Estland	19	2	9.52	14	4	22.22
Greenville-0	11	1	8.33	-	-	-
Isenberg	-	-	-	14	7	33.33
Kaunas-0	-	-	-	20	4	16.67
Kendalville	53	1	1.85	-	-	-
Koln-59	-	-	-	24	0	0.00
Lanark-0	-	-	-	10	8	44.44
*Landsberg erecta*	348	15	4.13	28	4	12.50
Le Mans-2	-	-	-	19	5	20.83
Limeport	-	-	-	20	4	16.67
Muhlen-0	29	0	0.00	20	4	16.67
Niederzenz-0	36	0	0.00	21	0	0.00
Nossen-0	38	0	0.00	-	-	-
Oystese-0	-	-	-	19	5	20.83
Poppelsdorf-0	-	-	-	20	4	16.67
RLD1	30	1	3.23	-	-	-
S96	37	1	2.63	-	-	-

^1^ No detectable host response after inoculation with *P. sojae* spores. ^2^ Visible necrosis at the inoculation site was observed.

### *PSS1* is required for nonhost resistance of Arabidopsis against *P. sojae*

Forty-two F_2:3_ families developed from the cross between *pss1* and Nd-0 were evaluated for segregation of host responses to the pathogen infection. At least 24 progenies of each F_2_ plants were scored for disease phenotypes. The segregation of alleles at the *PSS1* locus among the F_2:3_ families fit to the 1:2:1 genotypic ratio for a single gene model (*p* = 0.81; Table 2). This observation suggested that *PSS1* is a single gene with no apparent epistatic effect from *PEN1***.**

**Table 2:** **Segregation of *Pss1 *alleles among the F**_**2:3**_**families derived from a cross between the *pss1 *mutant and the ecotype Nd-0**

**Genotype**	**Observed**	**Expected**
Homozygous resistant (*Pss1Pss1*)	12	10.5
Heterozygous (*Pss1pss1*)	21	21
Homozygous susceptible (*pss1pss1*)	9	10.5
Total	42	42
***χ***^**2**^**value**	0.43	
***P*-value**	0.81	

**Figure 3:**
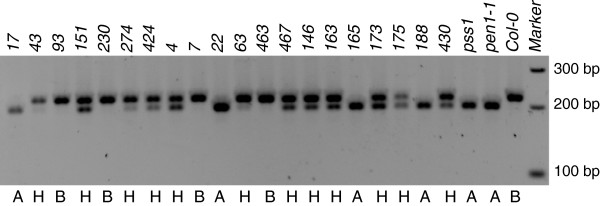
**Segregation of *PEN1 *alleles among 20 F**_**2:3**_**families homozygousfor *pss1.*** dCAPS marker based on SNP between *PEN1* and *pen1-1* alleles was used to determine the genotypes for alleles of the *PEN1* locus. Genotype A: homozygous for the *pen1-1* allele, B: homozygous for the *PEN1* allele, H: heterozygous.

In addition to these 42 F_2:3_ families, we determined the phenotypes of additional families. In this experiment, only eight progenies per family were screened to identify the F_2:3_ families that carry *pss1* in homozygous condition. To further confirm that *PSS1* is a single gene with no epistatic effect from *PEN1*, we evaluated the segregation of the *PEN1* alleles among 20 F_2:3_ families, homozygous for the *pss1* allele, using the dCAPS marker for *PEN1* alleles [7]. *PEN1* alleles segregated in a 1:2:1 ratio (*p* = 0.67) among the 20 families, homozygous for the *pss1* allele (Figure 3). This result suggested an independent segregation for the two genes. Among the 20 homozygous families for the *pss1* allele, four were shown to carry the *PEN1* allele in homozygous condition. If the *PEN1* allele was epistatic to *PSS1* and *PSS1* were to encode only a post-invasive resistance mechanism, then the *pen11* allele should have been in recessive homozygous condition among the *pss1* homozygous families. Thus, *PSS1* encodes a new form of penetration resistance.

### Expression of *P. sojae* effector genes in *pss1* during infection

**Figure 4:**
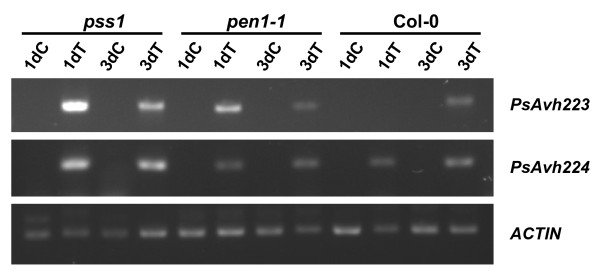
**Induction of the effector genes in the Arabidopsis and *P. sojae *interactions.** Expression levels of two *P. sojae* effector genes, *PsAvh223* and *PsAvh224*, highly induced in the soybean-*P. sojae* interaction were determined in an RT-PCR experiment. Detached leaves of *pss1, pen1-1* and Col-0 were inoculated with *P. sojae* or treated with sterile water droplets. The cDNA samples were used to amplify the two effector genes of *P. sojae* and Arabidopsis actin gene. Enhanced expression of both effector genes were observed in *pss1* but not in *pen1-1* and Col-0. 1dC, 1 day post water droplet treatment of detached leaves; 3dC, 3 days post water droplet treatment of detached leaves; 1dT, 1 day post inoculation with *P. sojae* zoospores; 3dT, 3 day post inoculation with *P. sojae* zoospores. Actin was used as an internal control.

To determine the extent of *P. sojae*-gene expression, we selected two effector genes to conduct RT-PCR. It has been shown that *P. sojae* carries over 370 candidate effector proteins containing N-terminal RXLR-dEER motifs [31]. We studied the expression of *PsAvh223* and *PsAvh224*[32] in *pss1*, *pen11* and Col-0 following inoculation with *P. sojae.* Both effector *P. sojae* genes were highly expressed in the *pss1* mutant as compared to *pen11* and Col-0 (Figure 4). This result indicates that the *P. sojae* colonized to a greater extent in *pss1* as compared that in *pen1-1* or Col-0.

### Mapping of the *PSS1* gene

In order to map the *PSS1* gene, we applied bulked segregant analysis (BSA) [33]. Four bulks of *P. sojae* susceptible plants each carrying 7–8 F_2:3_ susceptible families and one bulk of *P. sojae* resistant plants containing two homozygous (*PSS1PSS1*) and six heterozygous (*PSS1pss1*) F_2:3_ families were generated. These five bulks and Col-0 and Nd-0 were included in BSA. We used sequence-based polymorphic (SBP) [34], SSLP and CAPS markers in conducting BSA.

**Figure 5:**
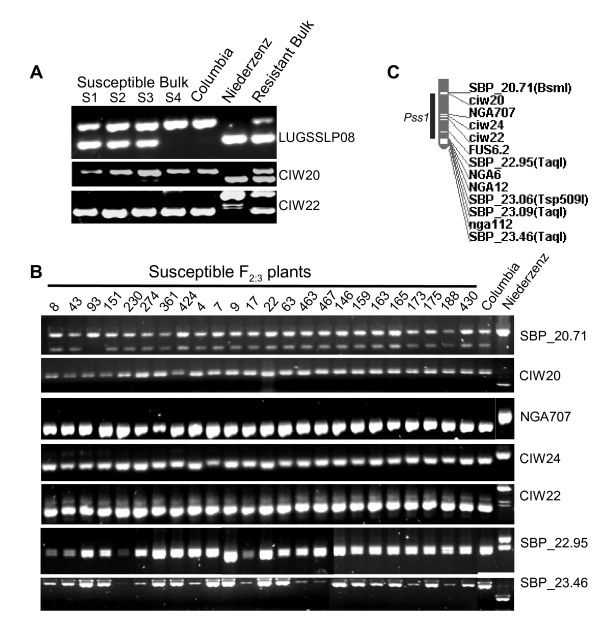
**Molecular mapping of the *PSS1 *locus. A,** Identification of SSLP markers linked to *PSS1*. Similar amplification patterns of SSLP markers CIW20 and CIW22 in susceptible bulks (S1, S2, S3 and S4) and Col-0 suggested that *PSS1* is putatively linked to the two markers. Amplification patterns of a distantly mapped SSLP marker, LUGSSLP08 in the bulked DNA samples are shown as the control. **B,** Co-segregation of *PSS1* with six molecular markers of the south arm of chromosome 3. Twenty-two susceptible F_2:3_ families except one, F_2:3_ family 93, showed same amplification patterns as in Col-0 for these markers. F_2:3_ family 93 showed recombination between *PSS1* and SBP_20.71. **C,** Molecular map of the *PSS1* region. Five SBP markers were developed for the *PSS1* region that was mapped to the southern arm of chromosome 3.

The *PSS1* region was putatively mapped to the south arm of chromosome 3 (Figure 5A). To develop a high-density map of the *PSS1* region, five SBP markers from this region were generated. SBP_20.71 marker showed a recombination event with the *PSS1* locus in the F_2:3_ family 93 suggesting that *PSS1* is located south of this marker (Figure 5B). No recombination was observed between *PSS1* and SBP_23.46 marker, located at the telomeric end of chromosome 3 (Figure 5C). The physical distance between SBP_20.71 and SBP_23.46 is ~2.75 Mb.

### The Arabidopsis *pss1* mutant is infected by the fungal pathogen, *Fusarium virguliforme,* which causes sudden death syndrome in soybean

**Figure 6:**
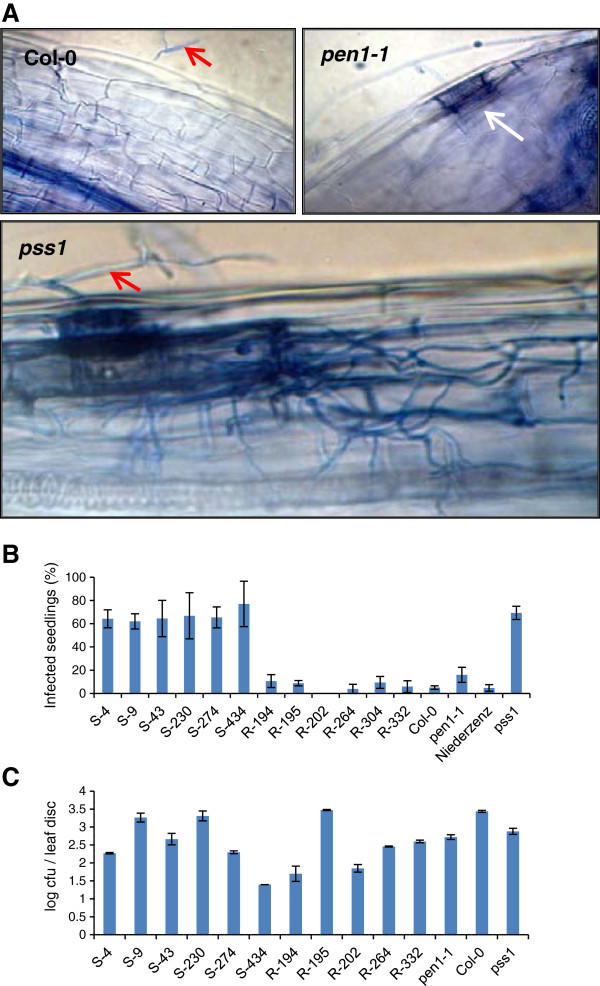
**The *pss1 *mutant was infected by fungal pathogen, *F. virguliforme*, but not by the bacterial pathogen, *P. syringae *pv. *glycinea. *A,** Response of *pss1* to *F. virguliforme* infection*.* Cell death and spread of mycelia stained with trypan blue were observed in infected seedlings of *pss1* but not in those of Col-0 or *pen1-1* following inoculation with *F. virguliforme* conidial spores. Single cell penetration by *F. virguliforme* was observed in *pen1-1* but not in Col-0 seedlings. Red arrows show the germinating conidia. White arrow shows a dead infected cell. All images were taken 2 days post- inoculation and at 400X magnification. **B,** Responses of six *P. sojae* susceptible (*pss1pss1*) (S-4 through S-434) and six resistant (*PSS1PSS1*) (R-194 through R-332) F_2:3_ families and the *pss1* mutant to inoculation with *F. virguliforme* conidial spores are presented. Data are the mean of three independent experiments. Error bars indicate S.E. among experiments. **C,** Response of *pss1* to *P. syringae* pv. *glycinea.* Disease response in colony forming units (cfu) of six *P. sojae* susceptible (*pss1pss1*) (S-4 through S-434) and five resistant (*PSS1PSS1*) (R-194 through R-332) F_2:3_ families and the *pss1* mutant 2 days following inoculation of intact leaves with *P. syringae* pv*. glycinea* are shown*.* Data are mean of three replications of a representative experiment. The experiment was repeated two times with similar results. Error bars indicate S.E. among experiments.

We investigated if *PSS1* controls Arabidopsis NHR against the fungal pathogen, *F. virguliforme* that causes sudden death syndrome (SDS) in soybean. From the segregating materials used for mapping the *PSS1* gene, we selected six F_2:3_ families that were homozygous for either *PSS1* or *pss1* alleles (Additional file 3) and used these families in determining the role of *PSS1* in NHR of Arabidopsis against *F. virguliforme*. Seedlings of the selected families were grown in 24-well microtiter plates for 10 days and then inoculated with *F. virguliforme* conidial spores. Infected seedlings were stained with trypan blue and observed under a light microscope (Figure 6A). Significant proportions of seedlings in six families carrying the *pss1* allele were infected by the fungal pathogen (Figure 6B). This result suggests that *PSS1* is also essential for NHR against the soybean pathogen, *F. virguliforme.*

### *PSS1* is not required for NHR of Arabidopsis against the non-adaptive pathogen *Pseudomonas syringae* pv. *glycinea* that causes bacterial blight in soybean

We investigated if *PSS1* is required for NHR of Arabidopsis against the bacterial pathogen, *Pseudomonas syringae* pv. *glycinea* (*Psg*) that causes bacterial blight in soybean [35]. We inoculated the six F_2:3_ families homozygous for *pss1* and five F_2:3_ families homozygous for the *PSS1* allele with *Psg* (Figure 6C). We observed no association of *PSS1* and *pss1* alleles with the colony forming units (cfu) of the bacterial pathogen. We classified the responses of the selected families into two broad groups, one with cfu comparable to those observed for Col-0 and Nd-0; and the other one with five- or more-fold lesser cfu as compared to those observed in Col-0 and Nd-0. Surprisingly, *pen1-1* consistently showed about 4-5-fold less bacterial growth as compared to that in Col-0 (Figure 6C). To determine if *PEN1* is required for growth of *Psg*, we genotyped the selected susceptible and resistant F_2:3_ families for the *PEN1* locus (Additional file 4). No association was observed between alleles at the *PEN1* locus and the levels of *Psg* cfu. These results suggested that an unknown mutation in the *pen1-1* genotype is most likely involved in enhancing resistance of Arabidopsis against *Psg* (Figure 6C) and the unknown gene could be a negative regulator of disease resistance.

## Discussion

Transfer of NHR mechanisms across species may lead to development of broad-spectrum and durable resistance in economically important crop species. Identification of *NHO1* and *PEN* genes established the molecular basis of NHR. It also suggested the feasibility of transferring single gene-encoded NHR across plant species for creating durable and broad-spectrum resistance [4,6-8].

Here we have described the Arabidopsis *PSS1* locus that carries one of the nonhost resistance genes conferring immunity of Arabidopsis against two important soybean pathogens, *P. sojae* and *F. virguliforme*. Considering the disease phenotypes observed in detached leaves of *pss1* as opposed to that in detached leaves of the *pen1-1* mutant following *P. sojae* inoculation (Figures 1 and 2), the NHR mechanism governed by *PSS1* is most likely important not only to provide penetration resistance, but also to confer necessary protection against further spread of the pathogen. *pss1* supports secondary hyphal growth and sporulation of *P. sojae* (Figure 2). These observations suggest that *PSS1* encodes a NHR defense mechanism that regulates both penetration and post-penetration resistance. It has been shown that the NHR mechanism at the post-haustorial stage is most important in sow thistle for providing resistance against a poorly adapted powdery mildew fungus, *Golovinomyces cichoracearum* UMSG1 [36]. Similar mechanism could also be important for NHR of Arabidopsis against the non-adapted oomycete pathogen, *P. sojae*.

Segregation data from a cross between *pss1* and Nd-0 revealed 1:2:1 genotypic segregation ratio for the alleles at the *PSS1* locus (Table 2); and therefore, it is a single gene. Alleles at the *PEN1* locus segregated independently of the alleles at the *PSS1* locus (Figure 3). The *P. sojae* susceptible phenotype of the *pss1* allele is manifested even in the presence of *PEN1*. Thus, *PSS1* controls a novel defense mechanism for penetration resistance against the oomycete pathogen, *P. sojae* and the fungal pathogen, *F. virguliforme*. *PEN* genes have been shown to regulate two distinct NHR mechanisms that are involved in penetration resistance. Monogenic inheritance of *PSS1* with no epistatic effect from PEN1 suggests that an additional Arabidopsis NHR mechanism is operative against penetration by oomycete and *Fusarium* pathogens. *PSS1* is located in an approximately 2.75 Mb region flanked by two sequence-based polymorphic markers, SBP_20.71 and the telomere-specific SBP_23.46 (Figure 5C). This region does not contain any characterized plant defense or disease resistance genes. Thus, most likely we have identified a novel nonhost resistance mechanism in Arabidopsis.

The important hallmarks of a successful adapted pathogen are its ability to establish feeding structures, derive nutrition from the host and finally to complete its lifecycle in the host plant [3]. Aniline blue staining has previously been used to show oomycete feeding structures such as runner hyphae [26]. We observed secondary hyphae even after 6 hpi suggesting that *P. sojae* is able to form feeding structures in *pss1* leaves at a very early stage following inoculation (Figure 2A). Sporangia are specialized asexual reproductive structures of oomycetes which can either germinate into hyphae or release about 10–30 zoospores to complete the asexual life-cycle. The male and female reproductive structures, antheridia and oogonia, are fused to develop oospores and complete the sexual life [37]. *P. sojae* developed both sporangia and oogonia in infected *pss1* leaves; and thus, completed its life cycle in this mutant (Figure 2B). In contrast, in *pen1-1* leaves the pathogen was able to penetrate single cells, which die following penetration; while in the wild type Col-0 leaves, germinated *P. sojae* zoospores failed to penetrate host cells (Figure 2B).

Lack of epistasis of *PEN1* on *PSS1* (Figure 3)*,* growth of secondary hyphae and rapid induction of effector genes in the *pss1* mutant, and most importantly completion of the *P. sojae*’s life cycle in infected *pss1* mutant leaves suggest that *PSS1* encodes a novel NHR mechanism that regulates both pre- and post-invasive resistance of Arabidopsis against the nonhost pathogen. Transfer of this to soybean could play an important role in creating broad-spectrum disease resistant not only against *P. sojae*, but also *F. virguliforme*. It is also possible that *PSS1* encoded resistance may be applicable to fighting diseases caused by oomycete pathogens in other crop species; such as potatoes and tomatoes.

It has been shown that lack of either of a functional pathway, the PEN1/SNAP33/VAMP721/722 or the indole- glucosinolates/metabolites pathway, involving the PEN2/PEN3 activity is sufficient to allow a non-adapted fungal pathogen to enter Arabidopsis mutant plants at a rate similar to that in an adapted host [38]. However, a complete loss of the subsequent post-invasion resistance mechanism encoded by plant defense genes *PAD4* and *SAG101* is necessary for a nonhost plant species to become a host for such non-adapted fungal pathogens [18]. In light of the critical role of the post-invasion genes as determinants of the nonhost status of Arabidopsis against non-adapted fungal pathogens, *PSS1*’s role at both pre- and post-haustorial levels in conferring NHR of Arabidopsis against *P. sojae* is novel.

*In vivo* trans-specific gene silencing in *Fusarium verticillioides* from transgenic tobacco provides molecular evidence suggesting a possible short biotrophic phase in *Fusarium species*[39]. *F. virguliforme* has been considered to be semi-biotrophic fungus with its ability to feed on live host soybean cells [40]. Thus, most likely *PSS1* may regulate the immunity against both hemibiotrophs, *P. sojae* and *F. virguliforme,* by using the same mechanism*.* The differing lifestyles of the two pathogens, *P. sojae* and *F. virguliforme* and the importance of *PSS1* in providing nonhost resistance against both of these pathogens hints at a crucial role of this gene in broader nonhost resistance of the model plant, Arabidopsis.

## Conclusions

Analyses of the segregants homozygous for alleles at both *PEN1* and *PSS1* loci revealed that PEN1 does not have any epistatic effect on the PSS1 function. The present study thus revealed a novel nonhost gene, *PSS1*, which confers immunity of Arabidopsis against two non-adaptive soybean pathogens, *P. sojae* and *F. virguliforme*. Responses of *pss1* and *pen1-1* to *P. sojae* invasion were distinct and PSS1 acts at both pre- and post-haustorial levels, while PEN1 acts at the pre-haustorial level. Identification and further characterization of the gene would provide us further insights about this new form of nonhost resistance against two non-adaptive soybean pathogens. This study thus laid the foundation for possible development of soybean germplasm with durable resistance against two serious pathogens.

## Methods

### Mutagenesis of *pen1-1*

About 15,000 *pen1-1* seeds were divided into three lots of ~5,000 seeds each. The three seed lots were then treated with 0.2%, 0.25%, and 0.3% EMS solution, respectively, for 15 h. The mutants were classified into three groups based on the concentration of EMS used for mutagenesis. Seeds were thoroughly washed 8 times in tap water and left in water on shaker for an additional hour. On an average, 1,000 seeds were sown on each flat (10-1/2" x 20-7/8"). Two weeks later plants were transplanted to trays containing 32 pots. The M_1_ plants were selfed and seeds of 3,556 M_2_ families were individually harvested.

### Inoculation methods and disease scoring

Two methods of inoculation were applied: i) seedling inoculation and ii) detached leaf inoculation. For the seedling inoculation, more than 70 *A. thaliana* seeds of individual M_2_ families were sterilized in the wells of 24-well microtiter plates (Costar® Corning Inc., Corning, NY) by first soaking in 70% ethanol for about 5 minutes and then washing with 50% Clorox bleach and 0.05% Triton X-100 for 10–15 minutes. The seeds were later rinsed four times with autoclaved water to remove any traces of bleach and/or ethanol. The seeds were then soaked aseptically in 300 μl autoclaved, double distilled water and incubated at 4°C for 48 h followed by incubation at 22°C for 10 days under constant light (100 μE/m^2^/s). Seedlings were then inoculated with 300 μl *P. sojae* zoospores race 25 (10^5^ zoospores/ml). After two days of incubation at 22°C in the dark, the inoculated seedlings were stained with trypan blue and then destained with saturated chloral hydrate for 48 h [23]. Destained seedlings were mounted on a glass slide in 50% glycerol and observed under a Zeiss microscope (Zeiss Incorporated, Thornwood, NY) and seedlings showing enhanced cell death in multiple cells were scored as susceptible.

For the leaf inoculation, the seeds were sown on LC1 soil-less mixture (Sun Gro Horticulture, Bellevue, WA) under a 16 h light/8 h dark regime at 21°C with approximately 60% relative humidity. The light intensity was maintained at 120–150 μE/m^2^/s [41]. Ten days after sowing, the seedlings were transplanted into a new LC1 mixture. The newly transplanted seedlings were covered with humidity domes for two days and thereafter watered every fourth day. A fertilizer mixture of 15:15:15::N:P:K (1% concentration v/v) was applied to the seedlings seven days after transplantation.

Three leaves (leaf # 4, 5 and 6 from the apex) were detached from 21-day old plants and placed on moist Whatman filter papers, in Petri dishes. Each leaf was then inoculated with 10 μl of *P. sojae* zoospore suspensions (10^5^/ml). The Petri dishes, following closing the lids, were incubated under constant light (50μE/m^2^/s) at 22°C. The inoculated plants were scored 48 and 72 h post inoculation (hpi) for resistant and susceptible host responses. In some experiments, 10-μl droplets of autoclaved double distilled water were placed on the surface of detached leaves as a negative control.

### Microscopic evaluations

Leaves of 21-day old Arabidopsis wild type Col-0, *pen1-1* and *pss1* mutant plants were inoculated with *P. sojae* spores (1.0 x 10^5^ spores/ml) and stained with trypan blue 7 days post inoculation (dpi) [23] and with aniline blue dye at 6 hours post inoculation (hpi) [25]. The stained leaves were mounted in saturated chloral hydrate for trypan blue dye [23] or in 70% glycerol and 30% aniline blue solution (0.01%) for aniline blue dye [25]. Stained images were examined using a Zeiss Axioplan II compound microscope equipped with AxioCam color digital camera.

### DNA preparation, PCR and BSA

Arabidopsis genomic DNA was extracted by CTAB method [42]. Young inflorescence or a rosette leaf was selected for DNA extraction. Equal amount (10 μg) of DNA from individual F_2:3_ families were mixed to obtain bulk DNA samples. The final DNA concentration of these bulk DNA samples for PCR was 20 ng/μl. The PCR reaction mixtures contained 2 mM MgCl_2_**(**Bioline, Taunton, MA), 0.25 μM each of forward and reverse primer (Integrated DNA Technologies, Inc., Coralville, Iowa), 2 μM dNTPs and 0.5 U Choice Taq polymerase (Denville Scientific, Inc., Metuchen, NJ). For SSLP markers, PCR was conducted at 94°C for 2 min, and then 40 cycles of 94°C for 30 s, 55°C for 30 s and 72°C for 30 s. Finally, the mixture was incubated at 72°C for 10 min. For the CAPS markers, PCR was conducted at 94°C for 2 min, and then five cycles of 94°C for 30 s followed by decreasing annealing temperatures from 55°C to 50°C (−1°C/cycle) and 72°C for 1 min. Then 40 cycles of 94°C for 30 s, 50°C for 30 s, and 72°C for 1 min were conducted. Finally, the reaction mixture was incubated at 72°C for 10 minutes. PCR was carried out in PTC-100 Programmable Thermal Controllers (MJ Research Inc.). The amplified products were resolved on a 4% agarose gel by running at 8 V/cm. The ethidium bromide stained PCR products were visualized following illumination with UV light.

### RNA isolation and RT-PCR experiments

Total RNA was isolated from leaf tissues using the TRIzol® reagent according to the manufacturer’s instructions (Invitrogen, Inc., Carlsbad, CA). RNA samples were treated with DNase I (Invitrogen, Inc., Carlsbad, CA) to eliminate any DNA contamination [43]. cDNAs were prepared according to manufacturer’s recommendations (Invitrogen, Inc., Carlsbad, CA). *PsAvh223, PsAvh224* and *AtACTIN-*specific primers (Table 3) were used to PCR amplify cDNA fragments from these samples. RT-PCR was conducted for the above genes using the cDNAs prepared from infected leaves at 1 d and 3 d post inoculation or treatment with water droplets. The following program was used to conduct PCR; 94°C 3 min and 35 cycles of 94°C for 30 sec, 60°C or 55°C and 72°C for 1 min followed by 72°C for 10 min. The transcripts of *AtACTIN* were simultaneously amplified for each set of RT-PCR reaction to show the possible variations in starting RNA amounts of different samples.

**Table 3:** Primers used in the RT-PCR experiment

**Gene**	^**1**^**Primer**	**Amplicon**
*PsAvh223*	F:GGCCACCCACACACCCCTCCCTCCCGTC	237
	R:CGGCGTCCTCGGCCTCGTCGTCTAG	
*PsAvh224*	F:GCGCGGCCTCGAGTTCCTTCTTCGTG	355
	R:CCTCCCTCCCGTCCGCTACAGTCATG	
*AtActin*	F:GGCGATGAAGCTCAATCCAAACG	491
	R:GGTCACGACCAGCAAGATCAAGACG	

^1^Primers: F, forward primer; R, reverse primer

### Molecular markers

Sequences of primers for SSLP markers were obtained from The Arabidopsis Information Resource (TAIR) database ( http://www.arabidopsis.org). SSLP markers, polymorphic between Col-0 and Nd-0, were selected to cover the entire genome with a density of one SSLP marker/2 Mb DNA. For the SSLP-thin regions, CAPS and SBP markers were designed [34]. The primers for the CAPS are presented in Table 4 and that for the SBP markers are presented in Table 5.

**Table 4:** List of CAPS markers polymorphic between Arabidopsis ecotypes Col-0 and Nd-0

**CAPS marker**	**^1^Restriction enzyme**	**^2^Primers**
1H1L-1.6	*Rsa* I, *Tsp*509I	F:CTAGAGCTTGAAAGTTGATG
		R:TTGAGTCCTTCTTGTCTG
20B4L-1.6	*Dde*I	F:CTAAGATGGGAATGTTGG
		R:GAACTCATTGTATGGACC
40E1T7	*Acc*I	F:GGTCCACTTTGATTCAAGAT
		R:GCAAGCGATAGAACATAACG
AF2	*Dde*I	F:TCGTCGTTTTTGTTTCCTTTTTCTTA
		R:CCATTCATTTAGGCCCCGACTTTC
B9-1.8	*Taq*I	F:CATCTGCAACATCTTCCCCAG
		R:CGTATCCGCATTTCTTCACTGC
CAT	*Taq*I, *Tsp*509I	F:GACCAGTAAGAGATCCAGATACTGCG
		R:CACAGTCATGCGACTCAAGACTTG
ER	*Dde*I	F:GAGTTTATTCTGTGCCAAGTCCCTG
		R:CTAATGTAGTGATCTGCGAGGTAATC
G4026	*Taq*I, *Rsa*I	F:GTACGGTTCTTCTTCCCTTA
		R:GGGGTCAGTTACATTACTAGC
G4711	D*de*I	F:CCTGTGAAAAACGACGTGCAGTTTC
		R:ACCAAATCTTCGTGGGGCTCAGCAG
GPA1.1	*Tsp*509I	F:ATTCCTTGGTCTCCATCATC
		R:GGGATTTGATGAAGGAGAAC
JM411	*Dde*I	F:GCGAACCACTAAGAACTA
		R:CTCGACTTTGCCAAGGAT
LFY3	*Rsa*I	F:GACGGCGTCTAGAAGATTC
		R:TAACTTATCGGGCTTCTGC
MI342	*Tsp*509I	F:GAAGTACAGCGGCTCAAAAAGAAG
		R:TTGCTGCCATGTAATACCTAAGTG
M555	*Acc*I	F:CCTTTAATTAGTTATCAAATC
		R:CTCTTGAATTATTAAGTTGACTAG
M59	*Rsa*I, *Tsp*509I	F:GTGCATGATATTGATGTACGC
		R:GAATGACATGAACACTTACACC
MBK23A	*Taq*I	F:GATGATTAGGCGCAAAATTGAG
		R:ATTACCAGCCTGGCTTCAGG
PAI1.1	*Taq*I, *Rsa*I, *Tsp*509I	F:GATCCTAAGGTATTGATATGATG
		R:GGTACAATTGATCTTCACTATAG
T20D161	*T*aqI, *Rsa*I, *Tsp*509I	F:CGTATTTGCTGATTCATGAGC
		R:ATGGTTTACACTTGACAGAGC
T6P5-4.8	*Rsa*I	F:TGAAAGACACCTGGGATAGGC
		R:CCAACTTTCGGGTCGGTTCC

^1^Restriction endonucleases used for individual CAPS markers are shown. ^2^Primers: F, forward primer; R, reverse primer

**Table 5:** **Sequence Based Polymorphic (SBP) markers generated for the*PSS1*region**

**Name**	^**1**^**Primer**	^**2**^**Enzyme**	**AmpliconSize (bp)**
SBP_22.95	F: GGAGGTTCCGTTACTC TTACTG R: CCACCGGAA GACGACGACTCTTC	*Rsa*I	309
SBP_22.98	F: CGACGTCACACTCTCC GTTA R: CCGATGATGGA GAAGGAAAA	*Taq*I	230
SBP_23.06	F: AAATTGGGGACACCA ACAAA R: GGTCCTCCTG GGAGAAAGAT	*Tsp*509I	180
SBP_23.09	F: TCGAATGATCCTTTCC TTTCA R: GCTTTTGCGA AAATGGGATA	*Taq*I	235
SBP_23.46	F: GACCAAATGTCTCTGA GATGTTC R: ACCCAAGG CGGTGTTGGCGAAAG	*Taq*I	520

^1^Primers: F, forward primer; R, reverse primer. ^2^Restriction endonucleases used for individual CAPS markers are shown

### Seedling inoculation with *F. virguliforme*

For inoculation of F_2:3_ families with *F. virguliforme,* more than 70 seedlings of each family were grown in 24-well microtiter plates (Costar® Corning Inc., Corning, NY) as described earlier. The seedlings of individual family were then inoculated with about 300 μl *F. virguliforme* spores (10^6^ spores/ml) and incubated in the dark for 48 h. The inoculated seedlings were then stained with trypan blue dye as previously described and observed under a microscope (Zeiss Inc., Thornwood, NY). Seedlings showing enhanced cell death in multiple cells were scored as susceptible.

### Leaf inoculation of F_**2:3**_ with the bacterial pathogen, *P. syringae* pv*. glycinea*

For leaf inoculation of F_**2:3**_ with *P. syringae* pv. *glycinea*, Arabidopsis plants were grown in a 10 h light/14 h dark period at 21°C under light intensity of 100–120 μmol/cm^2^/sec. *P. syringae* pv*. glycinea* was grown on King’s B medium containing rifampicin (100 μg/ml) at 28°C. For liquid culture, bacteria were grown in liquid King’s B medium without rifampicin at 25°C for 24 h. Four-week old plants were leaf inoculated with bacterial suspensions with 0.10 OD_600nm_ (~2 x 10^6^ cfu/ml) diluted in 10 mM MgCl_2_ solution [44]. Four leaves of each plant were inoculated on the abaxial side with 50 μl bacterial suspensions using the blunt end of a 1 ml syringe (BD, Franklin Lakes, NJ). Plants were then covered with a humidity dome until samples were harvested for plating. 1 cm diameter leaf discs from each inoculated leaf samples were harvested at 0 and 3 days post-inoculation. Leaf discs of eight leaves from two plants were pooled to make one replication and three biological replications were performed. Serial dilutions of the extracts from leaf disc samples were plated on King’s B medium containing rifampicin. Colony forming units (cfu) were counted 2 days following plating.

## Competing interests

The authors declare that they have no competing interests.

## Authors’ contributions

RS, BBS, MX and DS conducted the experimental work. RS and BBS wrote the first draft and contributed to writing the subsequent drafts of the manuscript. MKB conceived the research, designed the experiments and wrote the final draft of the manuscript. All authors read and approved the manuscript.

## Supplementary Material

Additional file 1 **EMS mutants created in*Arabidopsis thaliana pen1-1*mutants showed chlorophyll-lacking mutants among 5% of the M**_**2:3**_**families.** The albino seedlings are shown with arrows. Click here for file

Additional file 2 **A: Autoflourescene of*pss1*mutant leaf.** Detached leaves of 21-day old seedlings of the *pss1* mutant were mock inoculated with sterile water and stained with aniline blue and observed under ultraviolet epiflourescence 6 hours post inoculation. The image was taken at 50X magnification. The experiment was repeated three times with similar results. **B: The*pss1*mutant is a host for soybean oomycete pathogen,*P. sojae.*** Detached leaves of *pss1*mutant were inoculated with *P. sojae* zoospores (10^5^ spores/ml.) and stained with trypan blue dye 7 days post inoculation (dpi). Formation of sexual female reproductive structures, oogonia (oo) and asexual reproductive structures, sporangia (sp) indicate that the pathogen is able to complete its life cycle on the host *pss1* mutant leaves, thus signifying a complete breakdown of Arabidopsis nonhost resistance in this mutant. Numbers indicate the approximate size of the reproductive structures, which is in close agreement with the average size of the reproductive structures of the *Phytophthora* genus [45]. Click here for file

Additional file 3 **Identification of F**_**2:3**_**families homozygous for alleles at the*PSS1*locus. A,** Inoculation of a 10 day old *pss1* seedling with *P. sojae* spores followed by staining with trypan blue dye showed extensive hyphal growth and subsequent cell death. Image (100X magnification) was taken at 2 dpi. **B,** The indicated section of A at a higher magnification. **C,** Reponses of 10-day old seedlings of six F_2:3_ families, homozygous for the *pss1* allele (S-4 through S-434), and six F_2:3_ families, homozygous for the *PSS1* allele (R-194 through R-332), were inoculated with *P. sojae* zoospores. Data are mean of percent seedlings infected from three independent experiments. Error bars indicate standard error (S.E.) among experiments. Click here for file

Additional file 4 **Genotype of six*P. sojae*susceptible (*pss1pss1*) (S-4 through S-434) and five resistant (*PSS1PSS1*) (R-194 through R-332) F**_**2:3**_**families and the*pss1*mutant for the*PEN1*alleles.** A, homozygous for *pen1-1*, B, homozygous for *PEN1*; H, heterozygous. Click here for file

## References

[B1] Heath MC Implications of nonhost resistance for understanding host–parasite interactions 1985 In Genetic Basis of Biochemical Mechanisms of Plant Disease: APS Press

[B2] Heath MC The role of gene-for-gene interactions in the determination of host species specificity Phytopathology 1991 81 127 130

[B3] Thordal-Christensen H Fresh insights into processes of nonhost resistance Curr Opin Plant Biol 2003 6 4 351 357 10.1016/S1369-5266(03)00063-3 12873530

[B4] Kang L Li J Zhao T Xiao F Tang X Thilmony R He S Zhou JM Interplay of the Arabidopsis nonhost resistance gene NHO1 with bacterial virulence Proc Natl Acad Sci USA 2003 100 6 3519 3524 10.1073/pnas.0637377100 12626746 PMC152325

[B5] Lu M Tang X Zhou J-M Arabidopsis NHO1 is required for general resistance against Pseudomonas bacteria Plant Cell 2001 13 2 437 447 11226196 10.1105/tpc.13.2.437 PMC102253

[B6] Lipka V Dittgen J Bednarek P Bhat R Wiermer M Stein M Landtag J Brandt W Rosahl S Scheel D Pre- and postinvasion defenses both contribute to nonhost resistance in Arabidopsis Science 2005 310 5751 1180 1183 10.1126/science.1119409 16293760

[B7] Stein M Dittgen J Sanchez-Rodriguez C Hou B-H Molina A Schulze-Lefert P Lipka V Somerville S Arabidopsis PEN3/PDR8, an ATP binding cassette transporter, contributes to nonhost resistance to inappropriate pathogens that enter by direct penetration Plant Cell 2006 18 731 746 10.1105/tpc.105.038372 16473969 PMC1383646

[B8] Collins NC Thordal-Christensen H Lipka V Bau S Kombrink E Qiu J-L Huckelhoven R Stein M Freialdenhoven A Somerville SC SNARE-protein-mediated disease resistance at the plant cell wall Nature 2003 425 6961 973 977 10.1038/nature02076 14586469

[B9] Ellis J Insights into nonhost disease resistance: Can they assist disease control in agriculture? Plant Cell 2006 18 3 523 528 10.1105/tpc.105.040584 16513603 PMC1383630

[B10] Lipka U Fuchs R Lipka V Arabidopsis non-host resistance to powdery mildews Curr Opin Plant Biol 2008 11 4 404 411 10.1016/j.pbi.2008.04.004 18499508

[B11] Glazebrook J Contrasting mechanisms of defense against biotrophic and necrotrophic pathogens Annu Rev Phytopathol 2005 43 1 205 227 10.1146/annurev.phyto.43.040204.135923 16078883

[B12] Mellersh DG Heath MC Plasma membrane-cell wall adhesion is required for expression of plant defense responses during fungal penetration Plant Cell 2001 13 2 413 424 11226194 10.1105/tpc.13.2.413 PMC102251

[B13] Loehrer M Langenbach C Goellner K Conrath U Schaffrath U Characterization of nonhost resistance of Arabidopsis to the Asian soybean rust Mol Plant Microbe Interact 2008 21 11 1421 1430 10.1094/MPMI-21-11-1421 18842092

[B14] Jones JDG Dangl JL The plant immune system Nature 2006 444 7117 323 329 10.1038/nature05286 17108957

[B15] Schwessinger B Zipfel C News from the frontline: recent insights into PAMP-triggered immunity in plants Curr Opin Plant Biol 2008 11 4 389 395 10.1016/j.pbi.2008.06.001 18602859

[B16] Mittler R Herr EH Orvar BL van Camp W Willekens H Inze D Ellis BE Transgenic tobacco plants with reduced capability to detoxify reactive oxygen intermediates are hyperresponsive to pathogen infection Proc Natl Acad Sci USA 1999 96 24 14165 14170 10.1073/pnas.96.24.14165 10570216 PMC24208

[B17] Bittel P Robatzek S Microbe-associated molecular patterns (MAMPs) probe plant immunity Curr Opin Plant Biol 2007 10 4 335 341 10.1016/j.pbi.2007.04.021 17652011

[B18] Schulze-Lefert P Panstruga R A molecular evolutionary concept connecting nonhost resistance, pathogen host range, and pathogen speciation Trends Plant Sci 2011 16 3 117 125 10.1016/j.tplants.2011.01.001 21317020

[B19] Wrather JA Koenning SR Estimates of disease effects on soybean yields in the United States 2003–2005 J Nematol 2006 38 173 180 19259444 PMC2586459

[B20] Sandhu D Gao H Cianzio S Bhattacharyya MK Deletion of a disease resistance nucleotide-binding-site leucine-rich-repeat-like sequence is associated with the loss of the Phytophthora resistance gene Rps4 in soybean Genetics 2004 168 4 2157 2167 10.1534/genetics.104.032037 15611183 PMC1448712

[B21] Sandhu D Schallock KG Rivera-Velez N Lundeen P Cianzio S Bhattacharyya MK Soybean phytophthora resistance gene Rps8 maps closely to the Rps3 region J Hered 2005 96 5 536 541 10.1093/jhered/esi081 15958793

[B22] Burnham KD Dorrance AE VanToai TT St Martin SK Quantitative trait loci for partial resistance to Phytophthora sojae in soybean Crop Sci 2003 43 5 1610 1617 10.2135/cropsci2003.1610

[B23] Koch E Slusarenko A Arabidopsis is susceptible to infection by a downy mildew fungus Plant Cell 1990 2 5 437 445 2152169 10.1105/tpc.2.5.437 PMC159900

[B24] Bhattacharyya MK Ward EWB Expression of gene-specific and age-related resistance and the accumulation of glyceollin in soybean leaves infected with Phytophthora megasperma f. sp. glycinea Physiol Plant Pathol 1986 29 1 105 113 10.1016/S0048-4059(86)80042-X

[B25] Dietrich RA Delaney TP Uknes SJ Ward ER Ryals JA Dangl JL Arabidopsis mutants simulating disease resistance response Cell 1994 77 4 565 577 10.1016/0092-8674(94)90218-6 8187176

[B26] Adie BAT Perez-Perez J Perez-Perez MM Godoy M Sanchez-Serrano J-J Schmelz EA Solano R ABA is an essential signal for plant resistance to pathogens affecting JA biosynthesis and the activation of defenses in Arabidopsis Plant Cell 2007 19 5 1665 1681 10.1105/tpc.106.048041 17513501 PMC1913739

[B27] Bhadauria V Miraz P Kennedy R Banniza S Wei Y Dual trypan-aniline blue fluorescence staining methods for studying fungus-plant interactions Biotech Histochem 2010 85 2 99 105 10.3109/10520290903132196 19669979

[B28] Huang X Li J Bao F Zhang X Yang S A gain-of-function mutation in the Arabidopsis disease resistance gene RPP4 confers sensitivity to low temperature Plant Physiol 2010 154 2 796 809 10.1104/pp.110.157610 20699401 PMC2949010

[B29] Kwon Y Kim S Jung M Kim M Oh J Ju H Arabidopsis hot2 encodes an endochitinase-like protein that is essential for tolerance to heat, salt and drought stresses Plant J 2006 49 2 184 193 17156413 10.1111/j.1365-313X.2006.02950.x

[B30] Bell CJ Ecker JR Assignment of 30 microsatellite loci to the linkage map of Arabidopsis Genomics 1994 19 1 137 144 10.1006/geno.1994.1023 8188214

[B31] Jiang RHY Tripathy S Govers F Tyler BM RXLR effector reservoir in two Phytophthora species is dominated by a single rapidly evolving superfamily with more than 700 members Proc Natl Acad Sci USA 2008 105 12 4874 4879 10.1073/pnas.0709303105 18344324 PMC2290801

[B32] Wang Q Han C Ferreira AO Yu X Ye W Tripathy S Kale SD Gu B Sheng Y Sui Y Transcriptional programming and functional interactions within the Phytophthora sojae RXLR effector repertoire Plant Cell 2011 23 6 2064 2086 10.1105/tpc.111.086082 21653195 PMC3160037

[B33] Michelmore RW Paran I Kesseli RV Identification of markers linked to disease-resistance genes by bulked segregant analysis: a rapid method to detect markers in specific genomic regions by using segregating populations Proc Natl Acad Sci USA 1991 88 21 9828 9832 10.1073/pnas.88.21.9828 1682921 PMC52814

[B34] Sahu B Sumit R Srivastava S Bhattacharyya M Sequence based polymorphic (SBP) marker technology for targeted genomic regions: its application in generating a molecular map of the Arabidopsis thaliana genome BMC Genomics 2012 13 1 20 10.1186/1471-2164-13-20 22244314 PMC3323429

[B35] Huynh TV Dahlbeck D Staskawicz BJ Bacterial blight of soybean: Regulation of a pathogen gene determining host cultivar specificity Science 1989 245 4924 1374 1377 10.1126/science.2781284 2781284

[B36] Wen Y Wang W Feng J Luo M-C Tsuda K Katagiri F Bauchan G Xiao S Identification and utilization of a sow thistle powdery mildew as a poorly adapted pathogen to dissect post-invasion non-host resistance mechanisms in Arabidopsis J Exp Bot 2010 62 6 2117 2129 21193574 10.1093/jxb/erq406 PMC3060691

[B37] Tyler BM Phytophthora sojae: root rot pathogen of soybean and model oomycete Mol Plant Pathol 2007 8 1 1 8 10.1111/j.1364-3703.2006.00373.x 20507474

[B38] Lipka U Fuchs R Kuhns C Petutschnig E Lipka V Live and let die – Arabidopsis nonhost resistance to powdery mildews Eur J Cell Biol 2010 89 2–3 194 199 19963301 10.1016/j.ejcb.2009.11.011

[B39] Tinoco M Dias B Dall'Astta R Pamphile J Aragao F In vivotrans-specific gene silencing in fungal cells by in planta expression of a double-stranded RNA BMC Biology 2010 8 1 27 10.1186/1741-7007-8-27 20356372 PMC2907587

[B40] Iqbal M Yaegashi S Ahsan R Shopinski K Lightfoot D Root response to Fusarium solani f. sp. glycines: temporal accumulation of transcripts in partially resistant and susceptible soybean Theor Appl Genet 2005 110 8 1429 1438 10.1007/s00122-005-1969-9 15815926

[B41] Weigel D Glazebrook J A Laboratory Manual: Cold Spring Harbor Lab Press Arabidopsis 2002

[B42] Lukowitz W Gillmor CS Scheible W-R Positional cloning in Arabidopsis. Why it feels good to have a genome initiative working for you Plant Physiol 2000 123 3 795 806 10.1104/pp.123.3.795 10889228 PMC1539260

[B43] Krapp A Hofmann B Schafer C Stitt M Regulation of the expression of rbcS and other photosynthetic genes by carbohydrates: a mechanism for the 'sink regulation' of photosynthesis? Plant J 1993 3 6 817 828 10.1111/j.1365-313X.1993.00817.x

[B44] Mishina T Zeier J Bacterial non-host resistance: interactions of Arabidopsis with non-adapted Pseudomonas syringae strains Physiol plantarum 2007 131 3 448 461 10.1111/j.1399-3054.2007.00977.x 18251883

[B45] Werres S Marwitz R Veld W De Cock A Bonants PJM De Weerdt M Themann K Ilieva E Baayen RP Phytophthora ramorum sp nov., a new pathogen on Rhododendron and Viburnum Mycol Res 2001 105 1155 1165 10.1016/S0953-7562(08)61986-3

