# Supplementary figures and images for: Arabidopsis nonhost resistance gene PSS1 confers immunity against an oomycete and a fungal pathogen but not a bacterial pathogen that cause diseases in soybean
Source: BMC Plant Biol. 2012 Jun 13;12:87. doi: 10.1186/1471-2229-12-87 (PMC3507847; doi:10.1186/1471-2229-12-87)

## Slide 1
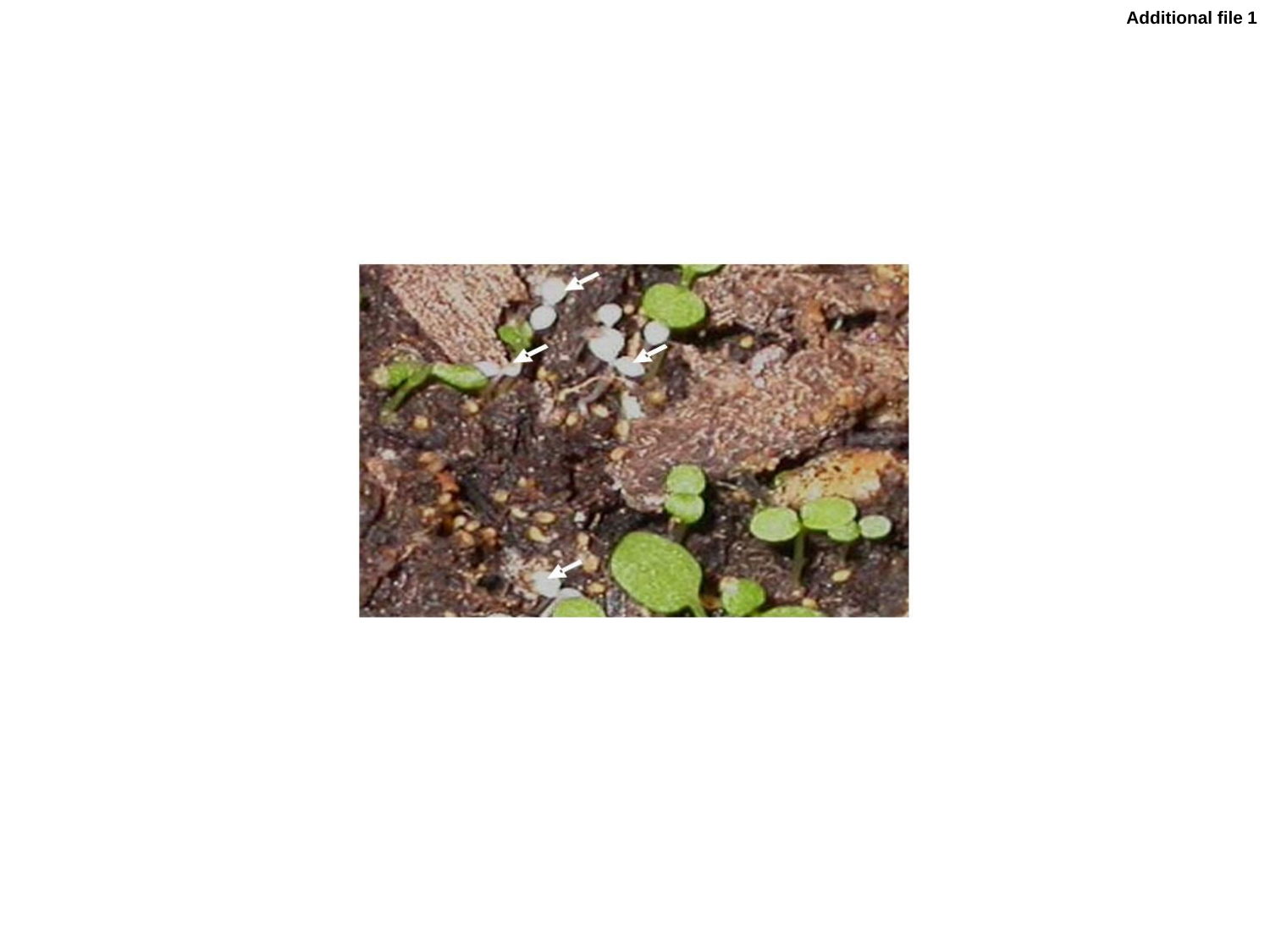

Additional file 1

Supplement: Additional file 1 — EMS mutants created inArabidopsis thaliana pen1-1mutants showed chlorophyll-lacking mutants among 5% of the M2:3families. The albino seedlings are shown with arrows. [file 1471-2229-12-87-S1.ppt]

## Slide 1
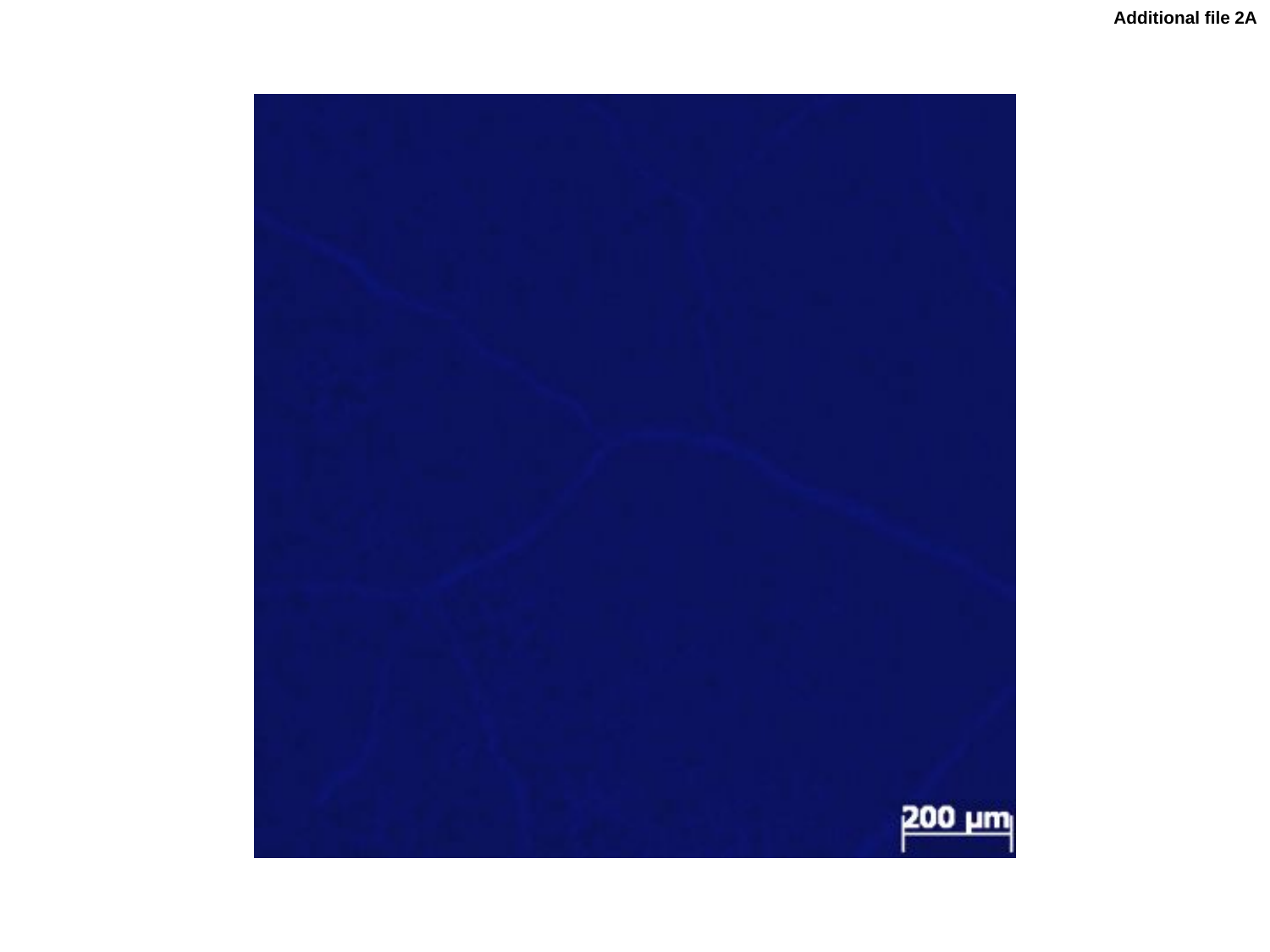

Additional file 2A

## Slide 2
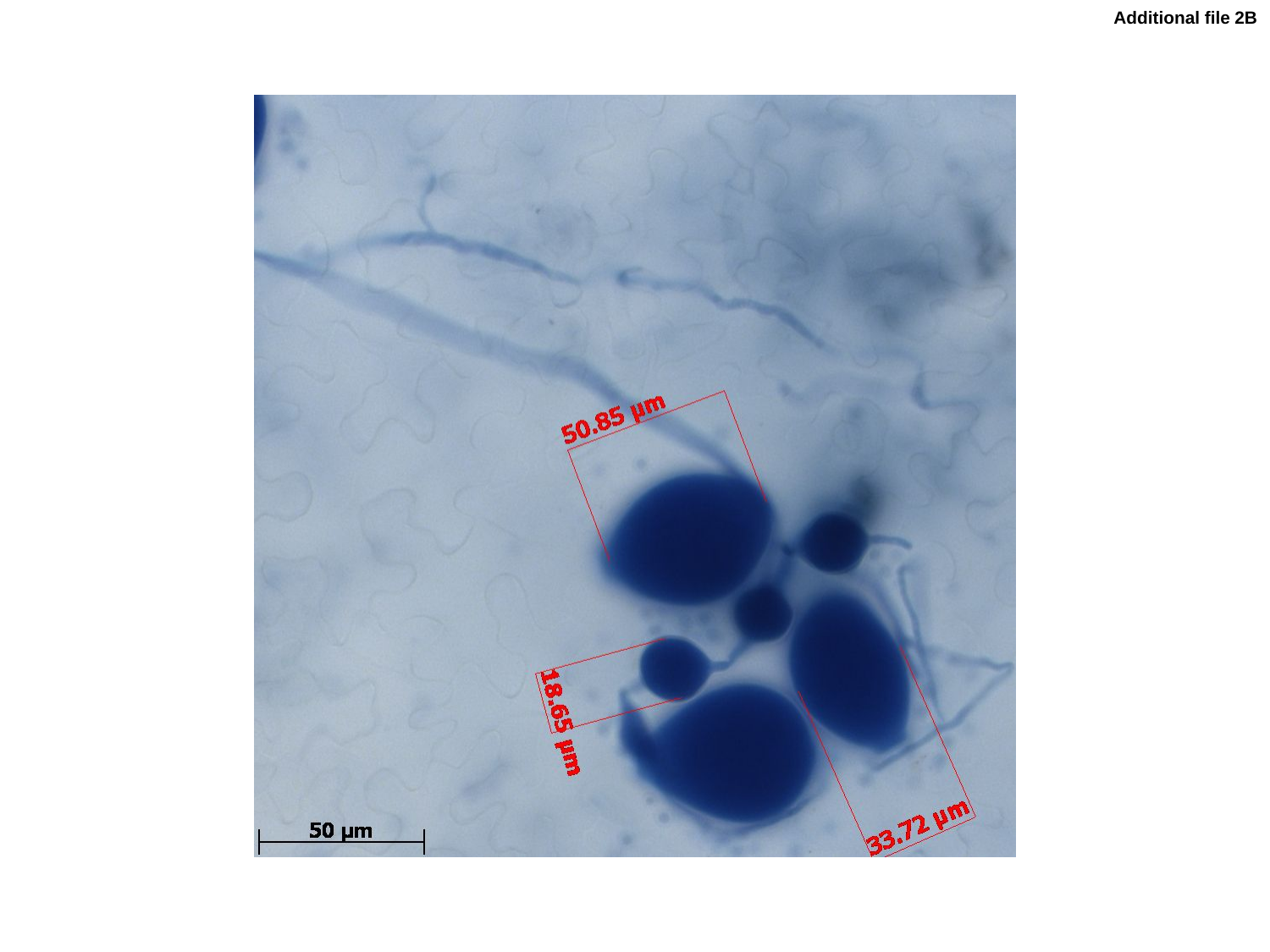

Additional file 2B

Supplement: Additional file 2 — A: Autoflourescene ofpss1mutant leaf. Detached leaves of 21-day old seedlings of the pss1 mutant were mock inoculated with sterile water and stained with aniline blue and observed under ultraviolet epiflourescence 6 hours post inoculation. The image was taken at 50X magnification. The experiment was repeated three times with similar results. B: Thepss1mutant is a host for soybean oomycete pathogen,P. sojae. Detached leaves of pss1mutant were inoculated with P. sojae zoospores (105 spores/ml.) and stained with trypan blue dye 7 days post inoculation (dpi). Formation of sexual female reproductive structures, oogonia (oo) and asexual reproductive structures, sporangia (sp) indicate that the pathogen is able to complete its life cycle on the host pss1 mutant leaves, thus signifying a complete breakdown of Arabidopsis nonhost resistance in this mutant. Numbers indicate the approximate size of the reproductive structures, which is in close agreement with the average size of the reproductive structures of the Phytophthora genus [45]. [file 1471-2229-12-87-S2.ppt]

## Slide 1
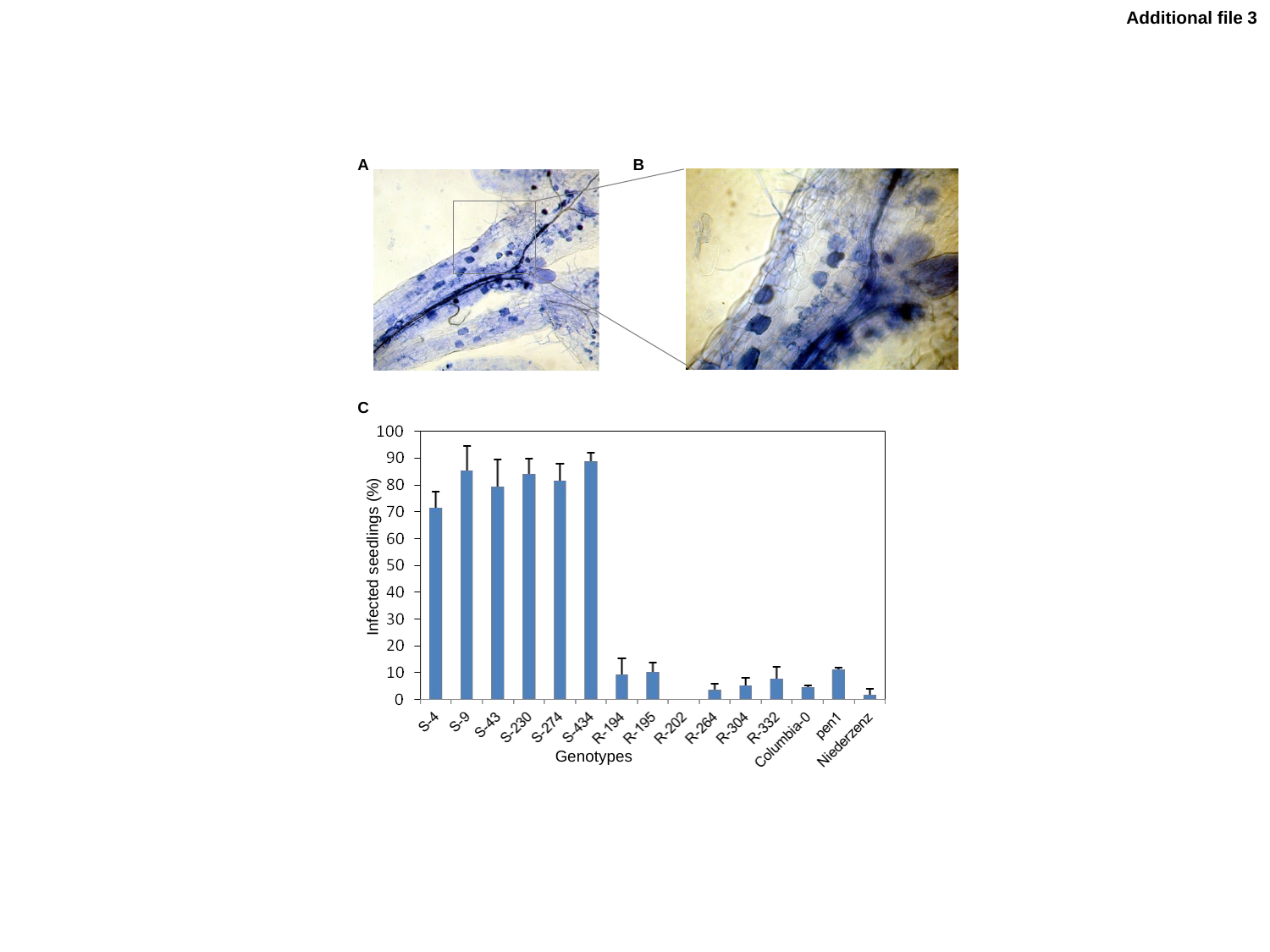

Additional file 3
A
B
C
Infected seedlings (%)
Genotypes

Supplement: Additional file 3 — Identification of F2:3families homozygous for alleles at thePSS1locus. A, Inoculation of a 10 day old pss1 seedling with P. sojae spores followed by staining with trypan blue dye showed extensive hyphal growth and subsequent cell death. Image (100X magnification) was taken at 2 dpi. B, The indicated section of A at a higher magnification. C, Reponses of 10-day old seedlings of six F2:3 families, homozygous for the pss1 allele (S-4 through S-434), and six F2:3 families, homozygous for the PSS1 allele (R-194 through R-332), were inoculated with P. sojae zoospores. Data are mean of percent seedlings infected from three independent experiments. Error bars indicate standard error (S.E.) among experiments. [file 1471-2229-12-87-S3.ppt]

## Slide 1
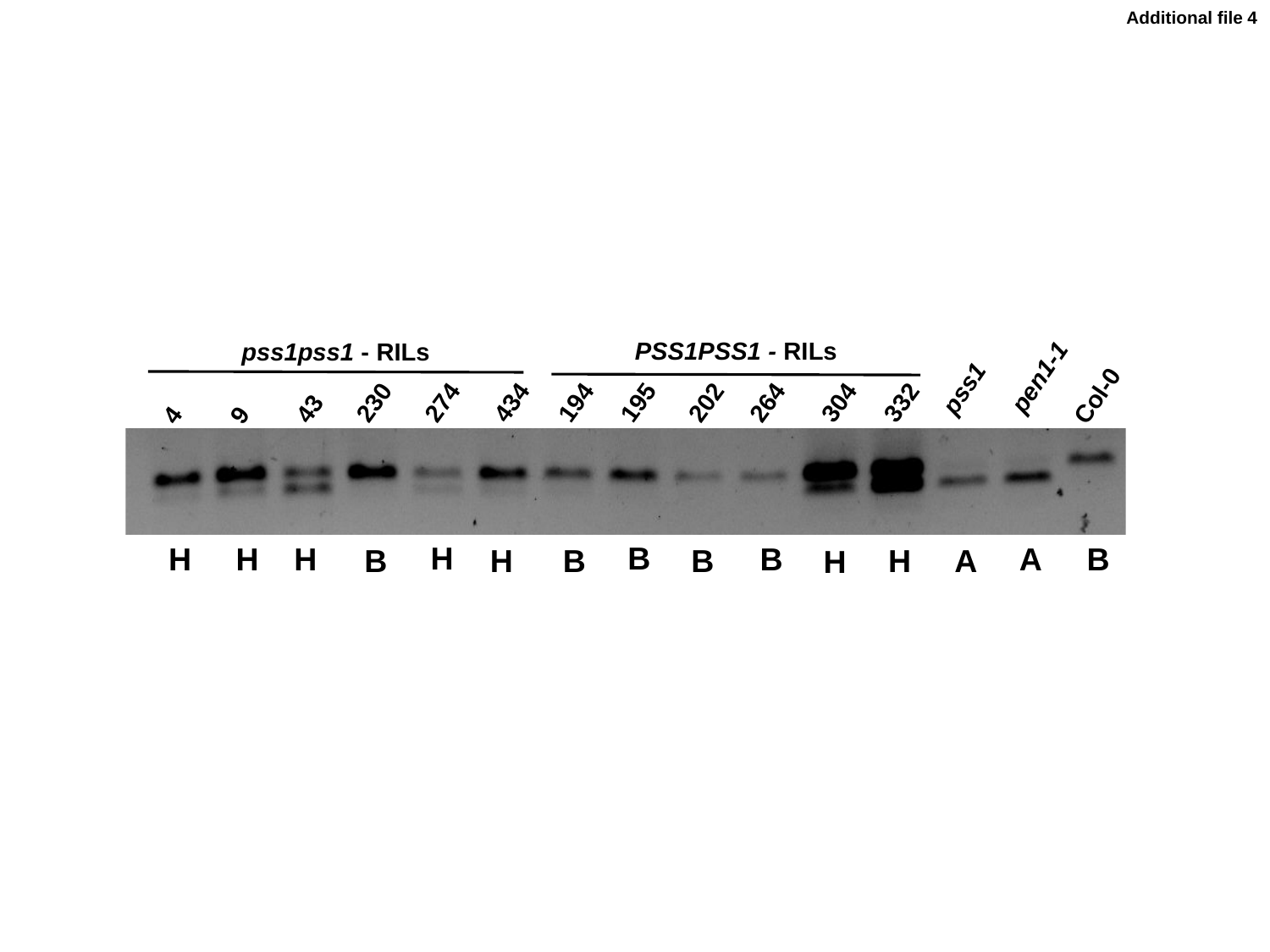

Additional file 4
PSS1PSS1 - RILs
pss1pss1 - RILs
pen1-1
pss1
Col-0
194
230
274
434
195
202
264
304
332
43
4
9
H
B
H
H
H
B
A
B
B
H
B
B
H
A
H

Supplement: Additional file 4 — Genotype of sixP. sojaesusceptible (pss1pss1) (S-4 through S-434) and five resistant (PSS1PSS1) (R-194 through R-332) F2:3families and thepss1mutant for thePEN1alleles. A, homozygous for pen1-1, B, homozygous for PEN1; H, heterozygous. [file 1471-2229-12-87-S4.ppt]
